# Analysis of gene variants in the GASH/Sal model of epilepsy

**DOI:** 10.1371/journal.pone.0229953

**Published:** 2020-03-13

**Authors:** Elena Díaz-Casado, Ricardo Gómez-Nieto, José M. de Pereda, Luis J. Muñoz, María Jara-Acevedo, Dolores E. López

**Affiliations:** 1 Institute of Neurosciences of Castilla y León, University of Salamanca, Salamanca, Spain; 2 Salamanca Institute for Biomedical Research, Salamanca, Spain; 3 Department of Cell Biology and Pathology, School Medicine, University of Salamanca, Salamanca, Spain; 4 Institute of Molecular and Cellular Biology of Cancer, CSIC.—University of Salamanca, Salamanca, Spain; 5 Animal facilities, University of Salamanca, Salamanca, Spain; 6 Sequencing Service-Nucleus, USAL and IBSAL, Salamanca, Spain; University of Modena and Reggio Emilia, ITALY

## Abstract

Epilepsy is a complex neurological disorder characterized by sudden and recurrent seizures, which are caused by various factors, including genetic abnormalities. Several animal models of epilepsy mimic the different symptoms of this disorder. In particular, the genetic audiogenic seizure hamster from Salamanca (GASH/Sal) animals exhibit sound-induced seizures similar to the generalized tonic seizures observed in epileptic patients. However, the genetic alterations underlying the audiogenic seizure susceptibility of the GASH/Sal model remain unknown. In addition, gene variations in the GASH/Sal might have a close resemblance with those described in humans with epilepsy, which is a prerequisite for any new preclinical studies that target genetic abnormalities. Here, we performed whole exome sequencing (WES) in GASH/Sal animals and their corresponding controls to identify and characterize the mutational landscape of the GASH/Sal strain. After filtering the results, moderate- and high-impact variants were validated by Sanger sequencing, assessing the possible impact of the mutations by “in silico” reconstruction of the encoded proteins and analyzing their corresponding biological pathways. Lastly, we quantified gene expression levels by RT-qPCR. In the GASH/Sal model, WES showed the presence of 342 variations, in which 21 were classified as high-impact mutations. After a full bioinformatics analysis to highlight the high quality and reliable variants, the presence of 3 high-impact and 15 moderate-impact variants were identified. Gene expression analysis of the high-impact variants of *Asb14* (ankyrin repeat and SOCS Box Containing 14), *Msh3* (MutS Homolog 3) and *Arhgef38* (Rho Guanine Nucleotide Exchange Factor 38) genes showed a higher expression in the GASH/Sal than in control hamsters. *In silico* analysis of the functional consequences indicated that those mutations in the three encoded proteins would have severe functional alterations. By functional analysis of the variants, we detected 44 significantly enriched pathways, including the glutamatergic synapse pathway. The data show three high-impact mutations with a major impact on the function of the proteins encoded by these genes, although no mutation in these three genes has been associated with some type of epilepsy until now. Furthermore, GASH/Sal animals also showed gene variants associated with different types of epilepsy that has been extensively documented, as well as mutations in other genes that encode proteins with functions related to neuronal excitability, which could be implied in the phenotype of the GASH/Sal. Our findings provide valuable genetic and biological pathway data associated to the genetic burden of the audiogenic seizure susceptibility and reinforce the need to validate the role of each key mutation in the phenotype of the GASH/Sal model.

## Introduction

Epilepsy is a complex neurological disorder, defined by sudden and recurrent seizures, which involves various underlying conditions associated with its etiology. Currently, epilepsy is one of the most common neurological diseases, affecting approximately 50 million people worldwide [1,2]. Although the causes of epilepsy are diverse and heterogeneous, some epilepsy syndromes are genetic and most often heritable conditions resulting from a pathogenic variant (mutation) with significant functional effects [3].

Genetic epilepsies can manifest as a primary syndrome caused by genetic alterations or as a secondary disorder of well-established metabolic or structural alterations, which may also have genetic causes [4]. Studies on genetic epilepsies have identified important channels and neurotransmitters for epileptogenesis, as well as syndromes or other genetic comorbidities, such as Fragile X Syndrome [5]. With the advent of whole genome sequencing (WGS), more complex genetic mechanisms underlying many forms of epilepsy are now being identified [6,7]. Whole exome sequencing (WES) makes it possible to identify alleles with direct functional consequences on proteins; indeed, most known human disease-causing variants reside within the exome [8,9].

In recent years, the number of known genes associated with epilepsy or syndromes with epilepsy has been increasing, to reach a total of 84 genes [4]. Many genetic epilepsies are caused by mutations in genes encoding ion channels (e.g., the voltage- gated sodium channel [10], genes involved in the synaptic vesicle cycle (e.g., *Stxbp1* [Syntaxin Binding Protein 1] [11]), and in different metabolic pathways, such as glucose transport (*Slc2a1* [solute Carrier Family 12 Member 1], [12]) and vitamin B6 metabolism, as well as genes encoding GABA (e.g. *Gabarg2* [Gamma-Aminobutyric Acid Type A Receptor Gamma2 Subunit], [13]) or NMDA (*Grin2a* [Glutamate Ionotropic Receptor NMDA Type Subunit 2A], [14]) receptors subunits. In addition to these genes directly related to epilepsy, in 2017, Wang and colleagues proposed another 73 genes associated with malformations in brain development and epilepsy, 536 genes associated with physical or other alterations accompanying epilepsy or seizures and 284 genes putatively associated with epilepsy [4].

Despite the numerous genetic variants identified in different types of seizures, few studies on genetic variants associated with audiogenic seizures have been conducted so far. Thus, genomic alterations have been partially addressed using DNA sequencing and immunohistochemistry in some of the mouse strains susceptible to audiogenic seizures, such as the Frings [15], Black Swiss [16] and DBA/2 [17] mouse models. Kaitsuka and colleagues [18] generated *Eef1d* (Eukaryotic Translation Elongation Factor 1 Delta) knockout mice that showed severe seizures in response to loud sounds, suggesting that *Eef1d* plays a key role in normal brain function, especially when exposed to external stimuli. Several other audiogenic models have been reported in the literature, including the Genetic Absence Epilepsy Rat from Strasbourg (GAERS) [19], the Wistar Albino Glaxo Rijswijk rat (WAG/Rij) [20] the Krushinsky-Molodkina rat [21], the genetically epilepsy-prone rats [22] and the Wistar Audiogenic Rat (WAR) [23], but their genetic alterations have not been described yet.

Animals models of audiogenic epilepsy are an invaluable tool for understanding the basic cellular mechanisms underlying epileptogenesis and for developing novel antiepileptic treatments. The genetic audiogenic seizure hamster from Salamanca (GASH/Sal) is a genetic model of audiogenic seizures derived from an autosomal recessive disorder. The GASH/Sal model exhibits generalized tonic-clonic audiogenic seizures induced by high-intensity acoustic stimulation, mimicking those sensory- evoked reflex seizures seen in humans [24]. This animal model of epilepsy has the potential to offer innate susceptibility to the development of convulsive seizures in response to acoustic stimuli, without genetic or chemical manipulation, in contrast to other models, which only enable epilepsy studies in the context of the effects of a knocked gene of interest.

In the GASH/Sal model, overexpression of the immediate early genes *Egr1* (Early Growth Response 1), *Egr2* (Early Growth Response 2) and *Egr3* (Early Growth Response 3) has been previously described [25]; however, little is known about the mutation responsible for susceptibility to seizures. We therefore characterized the underlying genetic variants of the GASH/Sal strain by WES, a high- throughput, cost-effective method for the identification of disease-causing mutations. For this reason, this approach is used in clinic research to study genetic causes of epilepsy [26–29].

In this study, the mutations identified were compared with those most commonly found in human epilepsy. Furthermore, the characterization of genetic predisposition and alterations caused by epileptic seizures allow the genetic audiogenic seizure models to be used for understanding the ictiogenic and epileptogenic processes [30,31].

## Material and methods

### Experimental animals

A total of 25 Syrian hamsters (*Mesocricetus auratus*) of 3 months of age were used in this study. Specifically,13 GASH/Sal animals from the inbred strain maintained at the vivarium of the University of Salamanca (USAL, Spain) and 12 wild-type Syrian golden hamsters (RjHan:AURA) from Janvier Labs (Le Genest-Saint-Isle, France), that were used as a control group.

The animals were maintained in Eurostandard Type III cages (Tecniplast, Italy), with Lignocel bedding (Rettenmaier Iberica), 14/10 light/dark cycle, 22-24^a^C room temperature with ad libitum access to food (Tecklad Global 2918 irradiated diet) and water.

In order to carry out the necessary experiments, all animals were deeply anesthetized under gas anesthesia (2.5% isoflurane), and the brains were removed quickly after decapitation.

All procedures and experimental protocols were performed in accordance with the guidelines of Commission Directive (2010/63/UE) for the care and use of laboratory animals and approved by the Bioethics Committee of the University of Salamanca (approval number 300). All efforts were made to minimize the number of animals and their suffering.

### Whole exome sequencing

Genomic DNA was extracted from brains of control and GASH/Sal hamsters using QIAamp DNA minikit (Qiagen, Hilden, GER, Europe). Total DNA quantitation and determination of quality was determined by NanoDrop-1000 measuring (Nano-Drop Technologies Inc., Wilmington, DE, EU). To sequence each sample, an individual indexed library was prepared. Genomic DNA was sheared into 200bp fragments using a Covaris M220 ultrasonicator. After the fragmentation process, end repair, A-tailing, adapter ligation and PCR reactions were performed using the SureSelect XT Library Prep Kit ILM for target enrichment (Agilent Technologies, Santa Clara, CA) according to manufacturer’s protocol. Purification after each step was performed using AMpure XP beads. For each sample library prepared, hybridization and capture were performed. The resulting genomic DNA (gDNA) libraries were hybridized to SureSelectXT Mouse All Exon library probes overnight at 65°C and captured with streptavidin magnetic beads, according to the manufacturer’s instructions. The SureSelect enriched DNA libraries were PCR-amplified to add an appropriate index to each sample. The concentration of the resulting indexed samples was determined by Qubit Fluorometer (Life Technologies, Grand Island, NY, USA) with the QubitTM dsDNA HS Assay Kit (Invitrogen). Fragment size distributions were assessed using a 4200 TapeStation System (Agilent Technologies, Santa Clara, CA).

Based on DNA concentration and average fragment size, libraries were normalized to an equal concentration of 4 nM and pooled by equal volume. Pooled libraries were diluted to a final concentration of 2.5 nM, including a 1% PhiX Control v3 spike-in. Sequencing was performed on an Illumina NovaSeq 6000 sequencer using the 300 cycles NovaSeq 6000 S2 reagent kit with paired-end, 2 x 150 base pair reads.

### Data processing and variant detection

Whole-exome sequence data from golden Syrian and GASH/Sal hamsters were processed at the Bioinformatics service of the University of Salamanca.

Quality control of the sequences was performed using the FASTQC software [32], assembling those that passed the checking process with the SPAdes program [33]. The sequence reads were aligned and compared with the reference genome MesAur1.0 (GCF 000349665.1) using the BWA software [34]. Variants were searched for as instructed in the manual of good practices of the GATK software [35]. These variants were functionally annotated, predicting their impact using the WEP software [36]. This annotation made it possible to use two filters. Filter 1: elimination of intergenic variants, of variants with a coverage of less than 1000 reads and of variants present in control samples. Filter 2: selection of moderate- and high-impact variants according to the WEP annotation and elimination of variants with a coverage of less than 1000 reads and of variants present in control samples.

High-confidence variants were selected after passing further variant filtering requirements and used in all downstream analyses. The following filtering requirements were used: a minimum coverage of 200 reads in control data and of 300 reads in GASH/Sal data, with a maximum of 1000 reads. The genes of interest were visually inspected for any possible duplicate.

We defined “qualifying variants” as those which met each of the following filtering criteria: 1) start-loss mutation or frameshift variant or splicing variant or stop-gain mutation or in-frame insertion or missense variant; 2) homozygous variants, because epilepsy is a recessive disease; 3) correct alignment; 4) correct coverage and 5) relation to epilepsy.

### Identification of altered pathways

All mutated genes were annotated with the corresponding pathway information using BioSystems Database [37], determining the number of genes containing missense, nonsense and frameshift mutations in each pathway of interest. Two-sided Fisher’s exact and hypergeometric tests were performed to identify functional enrichment of biological annotations. Pathway and Gene Ontology (GO) annotations were downloaded from the NCBI database (download date: 27/09/2018).

### Genotyping assay

To validate alterations in genes of interest, specific primers were designed for the genetic alterations of each gene using Primer-BLAST–NCBI–NIH available online (https://www.ncbi.nlm.nih.gov/tools/primer-blast/). The mutations were tested in the following genes: *Hrh4* (Histamine Receptor H4), *Msh3*, *Arfgef3* (ARFGEF Family Member 3), *Asb14*, *Orc5* (Origin Recognition Complex Subunit 5), *Arhgef38*, *Nup188* homolog (Nucleoporin 188), *Ttr* (Transthyretin), *Slc12a1*, *Zeb2* (Zinc Finger E-Box Binding Homeobox 2), *Kcnh7* (Potassium Voltage-Gated Channel Subfamily H Member 7), *Tsen54* (TRNA Splicing Endonuclease Subunit 54), *Grin2c* (Glutamate Ionotropic Receptor NMDA Type Subunit 2C), *Grik1* (Glutamate Ionotropic Receptor Kainate Type Subunit 1), *Btd* (Biotinidase), *Hesx1* (Hesx Homeobox1), *Cacna2d3* (calcium Voltage-Gate Channel Auxiliary Subunit Alpha2delta3) and *Cacna1a* (Calcium Voltage-Gate Channel Subunit Alpha1 A). All oligonucleotides used in this study were synthesized by ThermoFisher Scientific (Waltham, MA, USA). PCRs were performed using GoTaq Flexi DNA Polymerase (Promega, Madison, WI, USA), in a total volume of 50 μl, according to the manufacturer’s instructions. The following thermal cycling conditions were used for the PCR: 2 min at 95°C, followed by 35 cycles of 95°C for 30 sec, 60°C for 30 sec, and 72°C for 30 sec, with a final extension at 72°C for 5 min. After the PCR, 10 μl of 6X loading buffer (0.03% bromophenol blue, 60mM EDTA (Ethylenediaminetetraacetic acid), 10 mM Tris-HCl pH 7.6 and 30% glycerol) was mixed with the total volume (50 μl) of each PCR to separate the fragments by 2% agarose gel electrophoresis in 1X TAE (Tris acetate EDTA) (ThermoFishser Scientific) buffer for 30–40 min at 100 V. Nucleic acids on agarose gel were identified with RedSafe Nucleic Acid Staining Solution (iNtRON Biotechnology, Gyeonggi, South Korea) and photographed with an ultraviolet trans-illuminator. The DNA fragment of interest was excised from the agarose gel, purified using NucleoSpin Gel and PCR Clean-up (Macherey-Nagel, Düren, Germany), according to the manufacturer’s instructions, and eluted in 18 μl of DNase-free water. The samples were sequenced by Sanger sequencing at the DNASequencing Service-Nucleus from the University of Salamanca.

### Structural modeling of proteins

The Fold and Function Assignment System (FFAS) server [38] was used to search for homologs of the different study proteins in the Protein Data Bank (PDB). The structure of the altered regions was modeled using the program MODELLER [39]. Molecular figures were created using the program Pymol (The PyMOL Molecular Graphics System, Version 1.8 Schrödinger, LLC).

### Gene expression analysis

Quantitative real-time polymerase chain reaction (RT-qPCR) was used to determine mRNA expression. It is widely stablished that the inferior colliculus (IC), a critical integration center in the auditory midbrain pathway, is the epileptogenic focus in audiogenic seizure-prone models [40]. Therefore, the RT-qPCR analysis was carried out in the IC as the relevant nucleus that provide insights into the mechanism underlying epileptogenic processes. Total cellular RNA from the frozen IC of GASH/Sal and control hamsters was extracted using TRIzol Reagent (ThermoFisher Scientific) and electrophoresed in 1.5% agarose to check RNA integrity.

Total RNA was quantified by optical density at 260/280 nm and used to generate cDNA. For such purpose, the ImProm-II Reverse Transcription System (Promega) was used, according to the manufacturer’s instructions. Primers for genes of interest were designed using Primer-Blast (NCBI). Quantitative RT-PCR was performed using the standard curve method and Power SYBR Green RT-qPCR Reagents Kit (Applied Biosystems) in cycles of 95°C for 30s, 60°C for 1 min and 72°C for 30s. Differences in gene expression were described as fold-changes between GASH/Sal and control sample to identify changes in expression.

The primers were designed so that RT-qPCR products spanned two identified introns. Moreover, GAPDH was amplified with equal amounts of cDNA, which demonstrated identical GAPDH expression patterns in the inferior colliculus of control and GASH/Sal hamsters. Lastly, in all RT-qPCRs, RNA-free (negative) control samples produced no amplified products.

### Statistical analysis

Statistical analyses were carried out using the GraphPad Prism 6 software (GraphPad, Software, Inc. La Jolla, CA, USA). Data are expressed as the mean ± SD of 5 independent experiments per group. Unpaired t-test was used to compare differences between the experimental groups in the RT-qPCR results. A *p*-value of 0.05 was considered to be statistically significant.

To identify functional enrichment of biological annotations, both Fisher’s exact test and the hyper-geometric test were used to identify significantly overrepresented functional categories, with at least 3 annotated genes and a *p-*value < 0.05.

## Results

### Quality analysis of reads and alignments

All reads satisfactorily passed the quality control step, with an accuracy close to 99.9%. The alignment mapped a very high percentage of unique and paired reads (S1 Fig, and S1 and S2 Tables).

### Somatic mutations in the GASH/Sal model

After filtering, as described above, a total of 342 somatic mutations, which affected to 235 genes, were detected in all 3 GASH/Sal animals, including 21 high- and 321 moderate-impact mutations (S3 and S4 Tables, respectively). In addition, 235 genes were mutated in all samples. In terms of types of mutations, of the 321 moderate-impact mutations, 4 were in-frame deletions, 5 were in-frame insertions and 312 were missense variants. The high-impact variants were 11 frameshift variants; 1 frameshift, splicing and intron variant; 3 start-loss variants; 2 splice-site variants; 3 frameshift and splicing variants; 1 stop-gain mutation and in-frame insertion.

To identify gene variants possibly implicated in the development of audiogenic seizures in the GASH/Sal strain, additional selection criteria were defined and applied to focus our study on the variants that are most likely to be involved in the phenotype of our experimental model. These criteria include the presence of homozygous mutations in GASH/Sal animals, because susceptibility is inherited in an autosomal recessive inheritance pattern [24], the existence of similar mutations related to the development of seizures or different types of epilepsy, in both humans and animal models, and the lack of duplicates and adequate coverage. After applying these criteria, 22 qualifying variants were selected, namely 7 high- and 15 moderate-impact variants (Table 1). In the process, 7 high-impact variants were discarded because they corresponded to olfactory receptors, in addition to 1 variant of a vomeronasal receptor unrelated to epilepsy. As explained above, 4 high-impact variants were discarded because they were not homozygous mutations in the GASH/Sal. Lastly, the high-impact variants that were duplicated or lacked coverage were also discarded. Of the moderate-impact variants, 15 were selected by VarElect analysis (https://ve.genecards.org/#results) because they were more closely related to epilepsy (S5 Table).

**Table 1:** Selected gene variants.

Gene symbol	Protein encoded	Reference Genome	Position	Reference base	Alternative base	Consequence	Impact	Variation anotation (HGVS-nomenclature)	Average of reading containing the reference		Frequency of the altered allele	
		MesAur1.0 (GCF 000349665.1)						https://varnomen.hgvs.org/	Control	GASH/Sal	Control	GASH/Sal
***Arfgef3***	Brefeldin A-inhibited guanine nucleotide-exchange protein 3	NW_004801608.1	13637158	A	G	Splice acceptor variant	High	g.13637158 A>G	466.2	0.662	0.004	1
***Arhgef38***	*Rho guanine nucleotide exchange factor 38*	*NW_004801723*.*1*	*6139872*	G	A	Frameshift variant	High	p.(Q751fsTer13)	594.2	0.517	0.003	1
***Asb14***	*Ankyrin repeat and SOCS box protein 14*	*NW_004801678*.*1*	*6850319*	-	TT	Frameshift variant	High	p.(Y424FfsTer3)	800.3	2,899	0.004	0.998
***Btd***	*Biotinidase isoform X2*	*NW_004801678*.*1*	*5840054*	-	CTCCTC	Inframe insertion	Moderate	p.L15_G16insLL	706.0	0.376	0	1
***Btd***	*Biotinidase isoform X2*	*NW_004801678*.*1*	*5840096*	A	G	Missense variant	Moderate	p.D27G	637.2	0.428	0	1
***Btd***	*Biotinidase isoform X2*	*NW_004801678*.*1*	*5845773*	C	T	Missense variant	Moderate	p.S349L	322.0	0.264	0	1
***Cacna2d3***	*Voltage-dependent calcium channel subunit alpha-2/delta-3*	*NW_004801678*.*1*	*8959386*	T	C	Missense variant	Moderate	p.S677P	964.0	10,884	0	0.989
***Cacna1a***	*Voltage-dependent P/Q-type calcium channel subunit alpha-1A*	*NW_004801866*.*1*	*345894*	C	T	Missense variant	Moderate	p.A1034V	260.0	0.287	0	1
***Grik1***	*Glutamate receptor ionotropic*, *kainate 1*	*NW_004801646*.*1*	*9586732*	C	T	Missense variant	Moderate	p.H289Y	905.2	1,715	0.002	0.999
***Grin2c***	*Glutamate receptor ionotropic*, *NMDA 2C*	*NW_004801624*.*1*	*6526093*	T	C	Missense variant	Moderate	p.S499P	323.0	0.433	0	1
***Hrh4***	*Histamine H4 receptor isoform X1*	*NW_004801606*.*1*	*21265092*	A	G	Splice acceptor variant	High	g.21265092 A>G	993.0	0.989	0	1
***Jup***	*Junction plakoglobin*	*NW_004801871*.*1*	*459241*	G	C	Missense variant	Moderate	p.D729E	349.1	0.408	0.003	1
***Kcnh7***	*Potassium voltage-gated channel subfamily H member 7*	*NW_004801620*.*1*	*22955306*	C	G	Missense variant	Moderate	p.R972P	407.0	0.441	0	1
***Msh3***	*DNA mismatch repair protein Msh3*	*NW_004801607*.*1*	*21265092*	-	GGTGAATGCT	inframe insertion and stop gained	High	p.(I1011RfsTer1)	516.1	2,263	0.001	0.992
***Nup188 homolog***	*Nucleoporin NUP188 homolog*	*NW_004806193*.*1*	*1431*	G	A	Missense variant	High	p.V1I	541.2	2,995	0.004	0.998
***Orc5***	*Origin recognition complex subunit 5*	*NW_004801709*.*1*	*1435564*	-	AAGT	splice region variant	High	g.1435564insAAGT	385.0	1,497	0	0.998
***Ttr***	*Transthyretin; Thyroid hormone-binding protein*.	*NW_004801606*.*1*	*15489142*	G	A	Missense variant	Moderate	p.V85I	350.0	0.372	0	1
***Ttr***	*Transthyretin; Thyroid hormone-binding protein*.	*NW_004801606*.*1*	*15486349*	C	T	Missense variant	Moderate	p.P63L	566.0	2,423	0	0.995
***Hesx1***	*homeobox transcriptional repressor expressed in embryonic stem cells*	*NW_004801678*.*1*	*6939839*	T	C	Missense variant	Moderate	p.I93T	393.0	0.322	0	0.996
***Tsen54***	*tRNA-splicing endonuclease subunit Sen54*	*NW_004801624*.*1*	*5977082*	A	G	Missense variant	Moderate	p.Q377R	307.0	0.268	0	1
***Zeb2***	*Zinc finger E-box-binding homeobox 2*	*NW_004801620*.*1*	*5584410*	C	T	Missense variant	Moderate	p.P547S	996.0	0.998	0	1
***Slc12a1***	*Solute carrier family 12 member 1*	*NW_004801618*.*1*	*4813362*	T	G	Missense variant	Moderate	p.I13S	396.0	0.372	0	0.998

### Validation of high-quality variants

**Fig 1:**
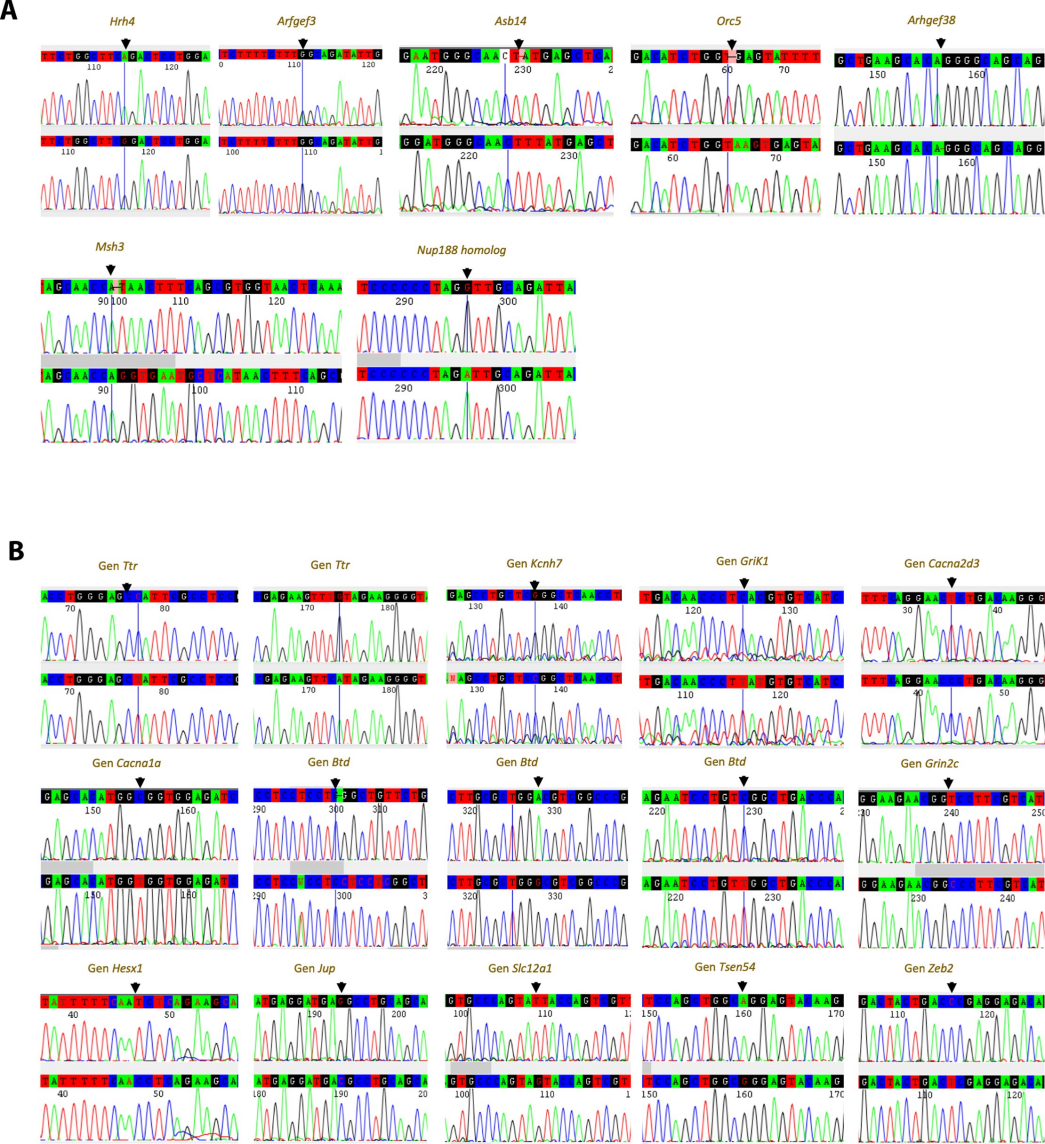
Validation of “high-quality mutations” by Sanger sequencing. A) Genes with high-impact mutations in GASH/Sal hamsters: in *Hrh4*, substitution of A by G in exon 3, position 22349951; in *Arfgef3*, heterozygous controls and GASH/Sal homozygous for position 13637158 in exon 15; in exon 6 of the *Asb14* gene, TT insertion in position 6850319; in the *Orc5* gene, AAGT insertion in position 1435564 in exon 6; in *Arhgef38*, substitution of AG by A in position 6139872; in *Msh3*, substitution of A by AGGTGAATGCTCA in position 21265094; in *Nup188 homolog*, substitution of G by A in position 1431. B) Genes with moderate-impact mutations in GASH/Sal hamsters: in the *Ttr* gene, two missense mutations, i.e., substitution of C by T in position 15486349 and substitution of G by A in position 15489142; in *Kcnh7*, substitution of C by G in position 22955306; in *Grik1*, substitution of C by T in position 9586732; in *Cacna2d3*, substitution of T by C in position 8959386; in the *Cacna1a* gene, substitution of C by T in position 345894; in the *Btd* gene, substitution of C by CCTCCTC in position 5840054, substitution of A by G in position 5840096 and substitution of C by T in position 5845773; in the *Grin2c* gene, substitution of T by C in position 6526093; in *Hex1*, substitution of T by C in position 6939839; in the *Jup* gene, substitution of G by C in position 459241; in *Slc12a1*, substitution of T by G in position 4813362; in the *Tsen54* gene, substitution of A by G in position 5977082; in *Zeb2*, substitution of C by T in position 5584410. All mutations were validated in 5 control and 5 GASH/Sal animals, showing the results from validations in one control animal and in one GASH/Sal animal by Sanger sequencing.

The 22 qualifying variants were confirmed by Sanger sequencing in 5 GASH/Sal and 5 Syrian control hamsters (Fig 1). After this validation, 4 of the 7 originally selected high-impact mutations were discarded (Fig 1A).

*Hrh4* has a predicted splice site mutation in position 22349951. However, analysis and validation of this mutation by Sanger sequencing demonstrate that the mutation located in exon 3 does not generate a splice acceptor variant (Hrh4- 201 ENSMAUT00000002722.1). This nucleotide change does not involve a missense mutation either.

The *Arfgef3* variant mutated in position 13637158, the *Orc5* variant mutation in position 1435564 and the *Nup188* homolog variant mutated in position 1431 were discarded because they are intronic variants.

The high-impact *Asb14*, *Arhgef38* and *Msh3* variants remained after filtering. All 15 moderate-impact mutations were also confirmed by Sanger sequencing (Fig 1B).

### The mutation in *Msh3* may change its interaction with DNA

**Fig 2:**
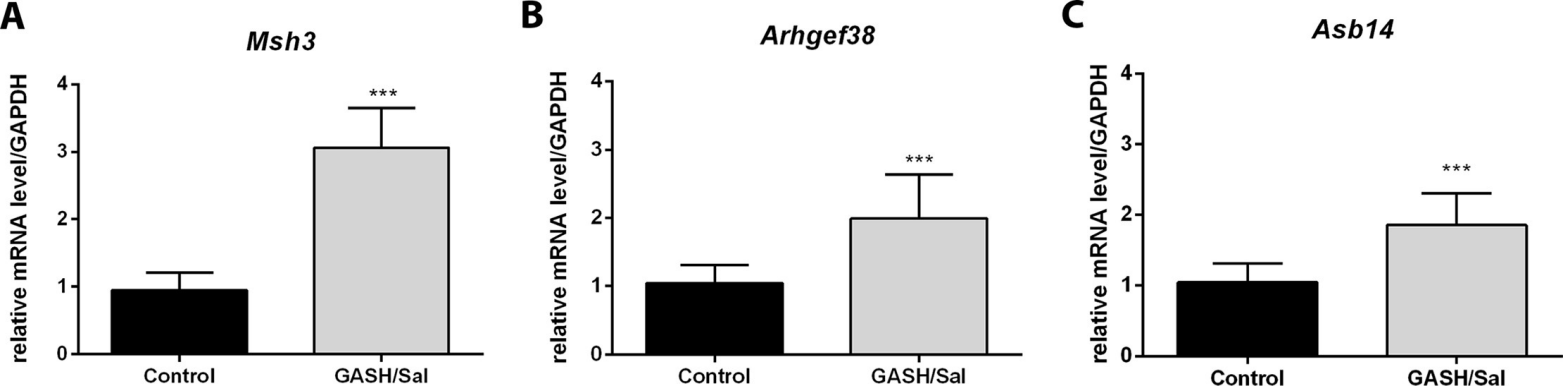
Variations in the expression of genes with high-impact exonic mutations selected in GASH/Sal. A) *Msh3* expression levels in GASH/Sal (n = 5) and control (n = 5) hamsters. B) *Arhgef38* expression levels in GASH/Sal (n = 5) and control (n = 5) hamsters. C) *Asb14* gene expression levels in GASH/Sal hamsters (n = 5). All three genes are significantly overexpressed in GASH/Sal animals in comparison with controls. Data are expressed as mean ± SD. ***p < 0.001; GASH/Sal versus control (Student’s t-test).

*Msh3* has a high-impact nonsense mutation and a moderate-impact point mutation K905N. *Msh3* gene expression analysis in the inferior colliculus of the GASH/Sal showed a significant increase in the expression of this gene in comparison with control hamsters (Fig 2A).

MSH3 forms, together with MSH2, the heterodimer MutSβ, which recognizes insertion-deletion loops and 3’ single-stranded overhangs in DNA. Several 3D structures of human MutSβ in complex with DNA have been reported [41]. The structures were solved using the fragment 219–1134 of human MSH3 (numbers of the Uniprot entry P20585). Only the region 225–1123 was ordered in the crystal structures, while short segments at the N- and C-termini were disordered. The 225–1106 region of human MSH3 is equivalent to the 183–1039 region of hamster MSH3 and they share 82% sequence identity. The region 1106–1137 of the human protein is not found in hamster MSH3, although part of this segment is disordered in the structure of human MSH3.

**Fig 3:**
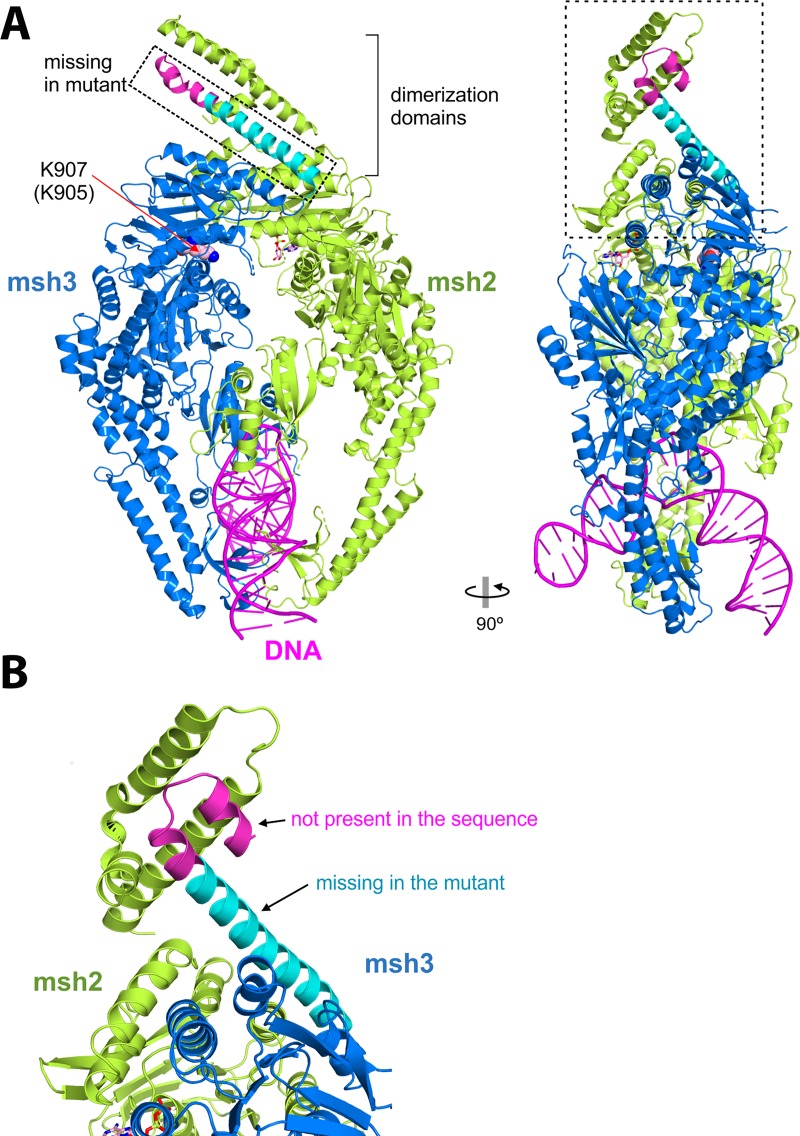
Structure of the human MutSβ complex bound to DNA (PDB 3THW). A) Two orthogonal views of the complex with MSH2 in green and MSH3 in blue, cyan and magenta. B) Detail of the region of the dimerization domains. The hamster sequence lacks the segment in magenta. The region that is equivalent to that lost in the truncated form is shown in cyan.

The deletion caused by the premature stop codon affects the C-terminal dimerization domain (Fig 3). Accordingly, the mutated form will likely be unable to form stable and functional dimers with MSH2, resulting in an inactive MutSβ complex.

On the other hand, the residue K905 is located on the surface of MSH3, facing the center of the dimer, albeit without contacting MSH2. The p.K905N substitution is unlikely to cause major structural alterations or to affect dimerization.

Lastly, neither the C-terminal nor the p.K905N mutation should directly affect the zone of interaction with DNA, although interaction with DNA should be strongly affected by changes in dimerization.

### The high-impact mutation of *Arhgef38* may prevent its function in protein-protein interactions

The *Arhgef38* gene has a high-impact variant with a frameshift mutation in exon 15. Gene expression analysis showed that the expression of this gene in the inferior colliculus is significantly higher in GASH/Sal than in control hamsters (Fig 2B).

**Fig 4:**
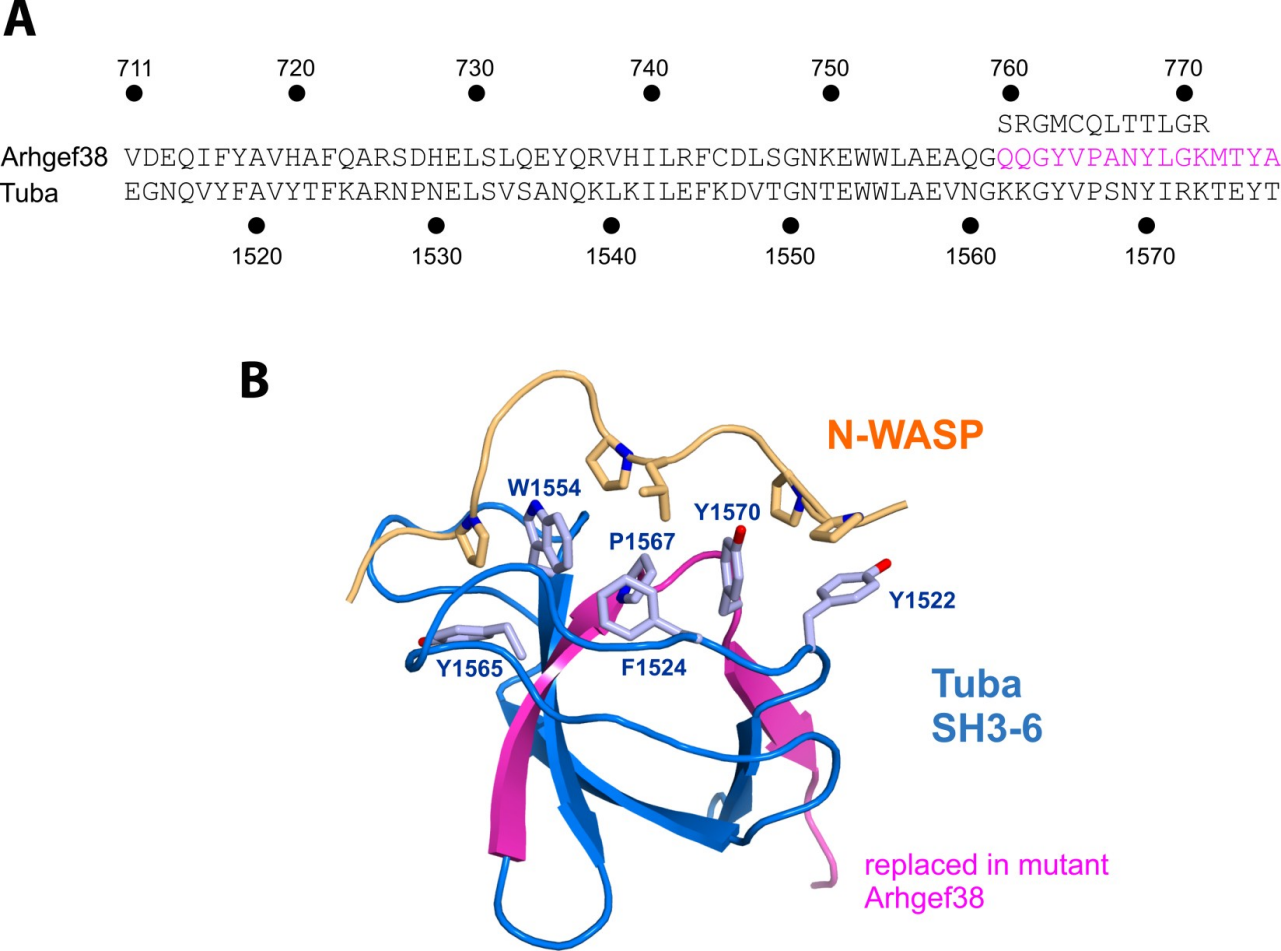
Structure of the SH3-6 domain of TUBA (ARHGEF36). A) Sequence alignment of the C-terminal SH3 domains of ARHGEF38 and TUBA. The region deleted in the mutant is indicated in magenta, and the sequence of the mutated protein is shown above. B) Representation of the structure of the SH3-6 domain of TUBA bound to a proline-rich region of N-WASP (PDB 4CC2), showing the side chains of some of the TUBA and N-WASP residues involved in the interaction. The altered region of mutant ARHGEF38 is highlighted in magenta.

The sequence of ARHGEF38 is similar to that of the human protein TUBA, also known as ARHGEF36 or Dynamin-binding protein. The TUBA sequence has 6 SH3 (SRC Homology 3) domains, with a Dbl-homology (DH) domain and a BIN/Amphiphysin/Rvs (BAR) domain between the fourth and fifth SH3 domains. The DH domain regulates the GTPase Cdc42, promoting guanine nucleotide exchange. ARHGEF36 and TUBA have 40% sequence identity in the sixth SH3 domain (SH3-6) (Fig 4A). Human ARHGEF36 is shorter than ARHGEF36 and only contains DH, BAR and the two SH3 domains of the C-terminal region.

The mutation affects the second SH3 domain (SH3-6 of TUBA), more specifically, the last two β sheets. Therefore, this sequence change likely causes a major structural change in this domain and the loss of its function. Most SH3 domains bind to proline- rich sequences. For example, the SH3-6 of TUBA binds to a proline-rich sequence of N- WASP (Neural Wiskott-Aldrich syndrome protein, Fig 4B). In summary, this mutation in the SH3-6 domain of ARHGEF38 likely causes its loss of function in protein-protein interactions, although no specific interactions mediated by this domain have been identified in ARHGEF38. Lastly, because the frameshift mutation likely results in an unstable SH3 domain, this misfolded conformation may lead to protein destabilization or aggregation.

### The mutation in *Asb14* may compromise its activity in proteasomal degradation

In GASH/Sal hamsters, *Asb14* has a frameshift mutation in codon Y429, which results in a premature stop codon (p.Y429X). Gene expression analysis showed significantly higher *Asb14* expression in the inferior colliculus of GASH/Sal hamsters than in that of control hamsters (Fig 2C).

**Fig 5:**
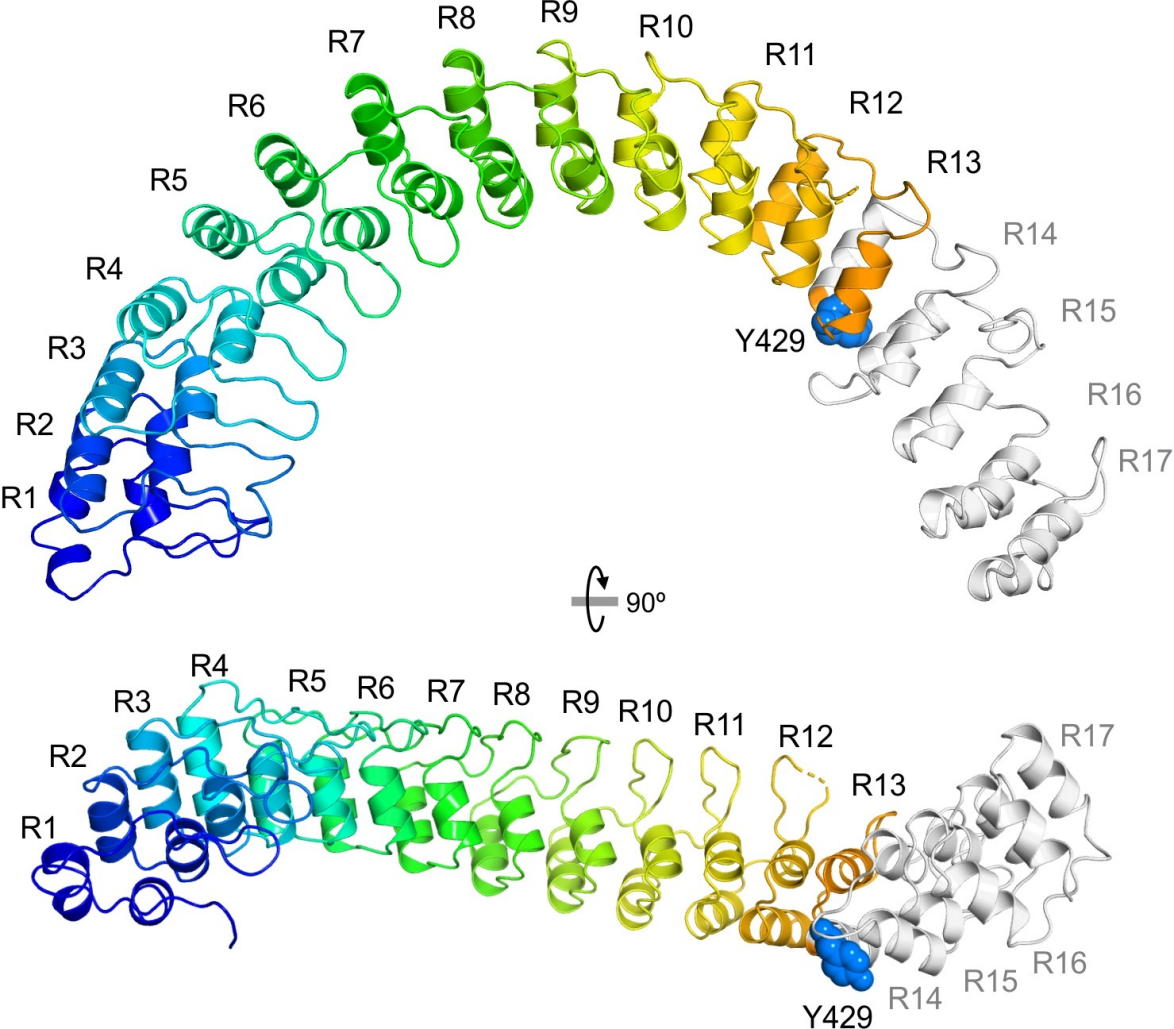
Structural model of the ankyrin repeat region of ASB14 (residues 1–556 of variant 1). The structure was modeled by homology using the crystal structure of Ankyrin B (PDB 4RLV).

The Fold and Function Assignment System (FFAS) server [42] was used to search for homologues of ASB14 in the Protein Data Bank (PDB). The top score was detected for human ankyrin-2 (also known as ankyrin-B). Then, the structure of the region encompassing residues 1–556 of ASB14 was modeled by homology using the structure of Ankyrin-2 as template (PDB entry 4RLV) [43] (Fig 5). The ASB14 region that spans from the N-terminus to residue 556 has 17 ankyrin repeats (R1 to R17). This domain of ASB14 is similar to the region of repeats R7 to R24 of ankyrin-2. Because this region of ASB14 consists in a series of juxtaposed repeats, it is likely to form a single structural unit.

Y429 is located between the two helices that form R13. The mutation creates a stop codon, which leads to a truncated protein with only the first 12 repeats. This truncated protein is most likely stable and viable because the R13-R17 region does not contribute directly to the structure of the R1-R12 region.

The function of the suppressors of cytokine signaling (SOCS) box domain, located in the C-terminal region, is to mediate interactions with the Elongin BC complex. Considering its similarity to other proteins with ankyrin repeats, this region of ASB14 may also mediate interactions with other proteins for their proteasomal degradation. Although the ASB14 protein truncated in the R1-R12 region is still able to interact with other proteins, this truncated form may have a dominant negative effect because the mutated form is no longer able to recruit the ubiquitin-protein ligase E3 complex. Alternatively, the loss of repeats R13-R17 may also compromise protein binding, in which case the truncated form would be inactive.

### Functional analysis of gene variants and altered pathways in the GASH/Sal model

**Fig 6:**
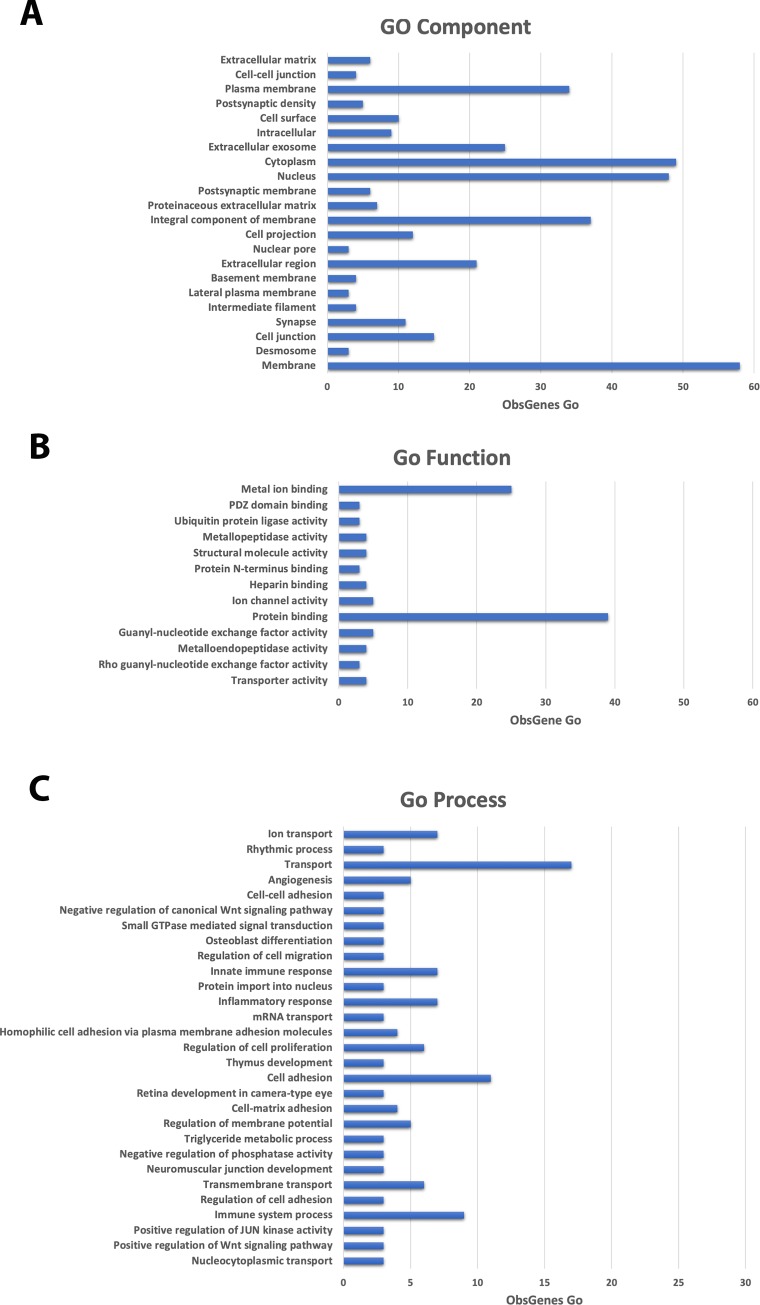
Gene ontology of gene variants. A) Cellular component. B) Molecular function. C). Biological process. Significant GO annotations are shown (*p* <0.001).

**Fig 7:**
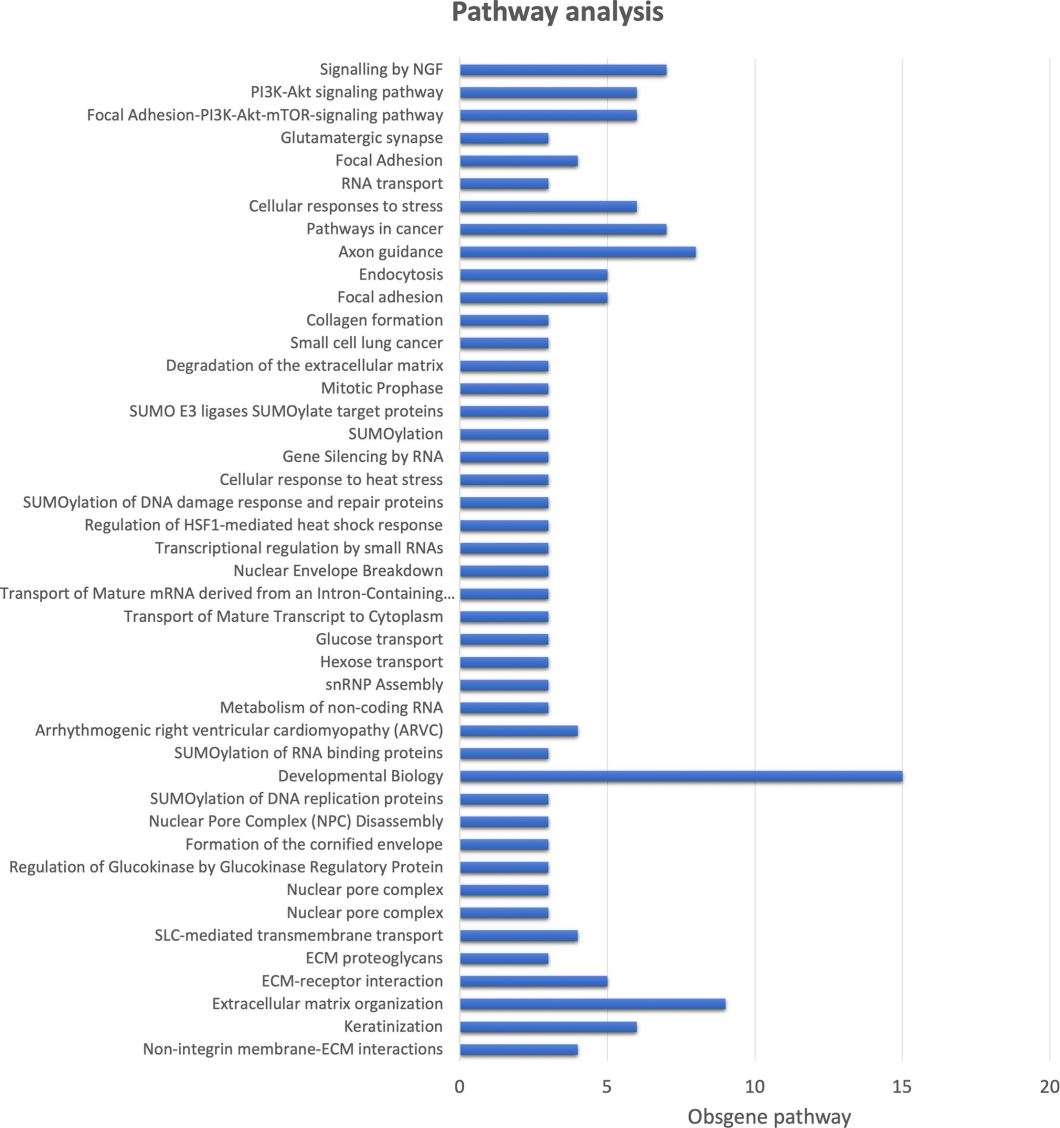
Gene set enrichment analysis using the KEGG, WikiPathways and REACTOME pathways.

Functional analysis of gene variants identified 22 GO components, 13 GO functions and 29 GO processes that were significantly altered (Fig 6). Biosystems analysis showed that 44 biological pathways were enriched in the list of significantly mutated genes by somatic mutation when analyzed using Fisher and hypergeometric tests (Fig 7). *Hrh4*, *Arfgef3*, *asb14*, *Orc5*, *Arhgef38* and the *Nup188* homolog genes with high-impact variants were not involved in any significantly altered pathway. *Msh3* was the only gene with a high-impact mutation that was involved in a significantly altered pathway, a cancer pathway (*p*-value = 0.0236). In this pathway, 6 other genes with moderate-impact mutations are involved: *Fgfr2* (Fibroblast Growth Factor Receptor 2), *Itga2b* (Integrin Subunit Alpha 2b), *Jup* (Junction Plakoglobin), *Lama1* (Laminin Subunit Alpha 1), *Lama3* (Laminin Subunit Alpha 3) and *Tpr* (Translocated Promoter Region, Nuclear Basket Protein). *Jup* also intervenes in a developmental biology pathway that is significantly altered (*p*-value = 0.0007). *Grin2c* gene, with moderate-impact mutation, is also involved in developmental biology and in 3 others significantly altered pathways: glutamatergic synapse (*p*-value = 0.0323), axon guidance (*p*-value = 0.0179) and NGF signaling (signaling by Neurotrophin receptors) (*p*-value = 0.0439). *Grik1* and *Cacna1a* play a role, together with *Grin2c*, in glutamatergic synapses. *Cacna2d3* is associated with arrhythmogenic right ventricular cardiomyopathy (*p*-value = 0.0008). *Slc12a1* is involved in SLC-mediated transmembrane transport (Solute carrier group of membrane transport proteins) (*p*-value = 0.0002). Lastly, other genes with moderate-impact mutations, such as *Ttr*, *Kcnh7*, *Btd*, *Hex1*, *Tsen54* and *Zeb2*, are not involved in significantly altered pathways.

## Discussion

In recent years, our understanding of the genetic basis of epilepsy has increased exponentially, highlighting the high complexity of inheritance patterns and genetic heterogeneity of epilepsy [44]. The study of genetic polymorphisms conveniently helps us understand the genetic bases of epilepsy by providing evidence of the involvement of one or several genes, thereby potentially clarifying pathogenic mechanisms. As an animal model of genetic audiogenic epilepsy, the GASH/Sal hamster can provide highly useful molecular data on this disease in humans, whenever the underlying mutations are similar.

Using a WES approach, we detected 21 high-impact mutations in the GASH/Sal model. After their analysis and validation, we selected three “high-quality mutations” which affect the *Msh3*, *ASb14* and *Arhgef38* genes. We focused our study on these mutations because they were the most likely to have severe effects on the encoded proteins and therefore on their function. The detected mutations in *ASb14*, *Arhgef38*, and *Msh3* lead to the expression of truncated proteins, which presumably were less stable than the wild-type forms. It is likely that the increased mRNA expression levels of the mutated genes resulted from a compensatory mechanism in cis that attempts to compensate the protein level and stability.

Recent studies have associated mutS homolog 3 (*Msh3*) variants, which play key roles in mismatch repair alongside other genes, with Lynch syndrome [45]. Our protein studies in GASH/Sal hamsters showed that the ability of the *Msh3* variant to bind and therefore repair DNA may be considerably impaired, and hence the increase in *Msh3* expression may be a cellular mechanism of compensation for its functional loss. Furthermore, *Msh3* is significantly enriched in cancer pathways, which further supports the hypothesis that this mutation is important for the GASH/Sal phenotype. However, no *Msh3* alteration directly related to any type of epilepsy has been published so far. Although the inability to form *Msh2* (Muts Homolog 2) dimers could affect DNA repair, the DNA repair function of *Msh2* is maintained even in patients with chronic intractable epilepsy who develop focal cortical dysplasia [46]. Protein analysis of the other two genes with high- impact variants, *Asb14* and *Arhgef38*, also predicts changes in their function, yet neither ASB14 (ankyrin repeat and SOCS box containing 14) nor ARHGEF38 have been associated with any type or case of epilepsy. However, a recent study has related alterations in the methylation pattern of the *Arhgef38* gene to changes in several pathways including axonal guidance signaling, calcium signaling, β-adrenergic signaling, and opioid signaling, in individuals with bipolar disorder [47].

Focusing on moderate-impact “high-quality variants”, we also identified mutations in GASH/Sal genes that were previously reported as polymorphisms associated with epilepsy, such as *Cacna1a*, *Cacna2d3*, *Grik1* and *Jup*.

The *Cacna1a* and *Cacna2d3* genes, which encode calcium voltage-gated channel subunit alpha1A and calcium voltage-gated channel auxiliary subunit alpha2delta3, respectively, are involved in different types of epilepsy, as described in the literature. *Cacna1a* S218L mice contain the missense mutation S218L in the alpha1a subunit of CaV2.1 voltage-gated Ca2+ channels and show increased excitatory neurotransmission and susceptibility to spreading depolarization. In addition, *Cacna1a* S218L mice homozygous for the mutation develop multiple, spontaneous, tonic clonic seizures and die from sudden unexpected death in epilepsy (SUDEP) [48]. Recently, Jiang and colleagues [49], have demonstrated that both gain- and loss-of-function *Cacna1a* mutations are associated with developmental epileptic encephalopathies.

In turn, *Grik1* encodes glutamate ionotropic receptor kainate type subunit 1. The key role these glutamate receptors play in excitatory neurotransmission and in synaptic plasticity suggests their participation in epileptogenesis [50]. In fact, different studies have associated polymorphisms in this gene with different types of epilepsy, including juvenile absence epilepsy (JAE), a common type of idiopathic generalized epilepsy [51], and SUDEP, the main cause of mortality related to epilepsy in young adults. In the latter, gene variants have been found in *Jup*, another gene with a variant validated in the GASH/Sal [52]. Conversely, other studies found no relationship between juvenile absence epilepsy and mutations in the coding sequence of the gene *Grik1* [53]. Discrepancies also occur in drug-induced animal models. No changes in *Grik1* expression are observed in animal models induced by pilocarpine administration, whereas *Grik1* is upregulated when inducing an injury in the CA1 subregion by a single injection of kainic acid in juvenile rats with previously sustained neonatal seizures [54, 55]. Moreover, *Cacna1a* and *Grick 1*, together with *Grin2c*, participate in the glutamatergic synapse pathway, which is significantly enriched in the GASH/Sal model.

Therefore, these genes have high potential for a direct role in the etiopathology of the GASH/Sal.

The GASH/Sal model also has a moderate-impact variant of the Biotinidase (*Btd*) gene. Biotinidase deficiency is a recessive autosomal disease characterized by neurological disorders, including seizures in 70% patients with this disorder. Given this high incidence, infants younger than one year with poorly controlled seizures should be referred for differential diagnosis [56].

Copy number variations (CNVs) are also considered a key factor in the etiology of epilepsy. A recent study based on array comparative genomic hybridization analyzed CNVs in epileptic patients and found a “BGNADP” motif in *Btd*, which could be a key motif in epilepsy [57]. Based on the relationships between *Btd* and epilepsy described in the literature, the variant of this gene in the GASH/Sal could also be important for its phenotype, although *Btd* is not involved in any significantly enriched pathway. Similarly, several studies have shown that heterozygous mutations of the *Zeb2* gene cause the Mowat-Wilson syndrome, which is characterized by seizures with onset in the second year of life, among other symptoms [58–62]. The gene *Zeb2* encodes the zinc finger E-box binding homeobox 2 protein. Nonsense mutations, frameshift mutations and deletions in this gene have been associated with the Mowat-Wilson syndrome [60,62]. However, we identified using WES, a moderate- impact missense mutation in the GASH/Sal hamster, which could be similar to another missense variant in *Zeb2* previously identified in a case of Early Infantile Epileptic Encephalopathy [63], thus expanding the range of phenotypes associated with variants in this gene, including the GASH/Sal phenotype.

Other genes with polymorphisms validated in the GASH/Sal, namely *Tsen54*, *Hesx1* and *SLC12a1*, are also related to epilepsy, albeit secondarily because polymorphisms in these genes are primarily associated with other diseases. Polymorphisms in *Tsen54* have been detected in pontocerebellar hypoplasia, a rare autosomal recessive neurodegenerative disease, in which epilepsy is found in approximately 50% patients [64–66]. *Hesx1* exon sequencing has revealed different mutations in patients with panhypopituitarism who present with hypoglycemic seizures [67]. *Slc12a1* encodes a renal-specific Na-K-2Cl cotransporter, and loss-of-function mutations in this gene are related to Bartter’s syndrome, an autosomal recessive form of metabolic alkalosis. Patients with this condition sometimes exhibit seizures [68–70].

Although these genes, according to the data on the functional consequences of their mutations and on the literature, could clearly be involved in the etiology of GASH/Sal seizures, other polymorphisms detected and validated as “high-quality mutations” cannot be overlooked. Such genes have not been previously associated with any type of epilepsy, but they intervene in pathways related to neuronal excitability and therefore may be functionally relevant in the pathogenesis of seizures in the GASH/Sal. This is the case of genes such as *Kcnh7* and *Grin2c*. *Kcnh7* is a member of the voltage- gated K+ channel Kv11 family and is above all expressed in the brain. Indeed, polymorphisms in this gene have been associated with different responses to pharmacological treatment with risperidone in patients with schizophrenia [28]. *Grin2c*, together with *Grin1* (Glutamate Ionotropic Receptor NMDA Type Subunit 1), *Grin2A*, *Grin2D* (Glutamate Ionotropic Receptor NMDA Type Subunit 2D), *Grin3A* (Glutamate Ionotropic Receptor NMDA Type Subunit 3A) and *Grin3b* (Glutamate Ionotropic Receptor NMDA Type Subunit 3B), encode different subunits of N-methyl-D-aspartate receptors (NMDARs). These receptors are involved in nerve transmission through glutamate, and their deregulation has been associated with disorders such as schizophrenia and autism. In this context, Yu and colleagues detected, *Grin2c* missense and frameshift mutations, among others, associated with susceptibility to schizophrenia [71].

No polymorphisms in the *Ttr* gene have been associated with epilepsy either, although other polymorphisms in the same gene have been linked to familial amyloid polyneuropathy with rapid deterioration [72]. The only relationship between *Ttr* and epilepsy in the literature is the immunopositivity for *Ttr* in patients with intracerebral amyloidoma (ICA), in which a patient was diagnosed with epilepsy and cognitive impairment [73].

Despite advances in the study of epilepsy, very little is known about genes altered in animal models of epilepsy, except for monogenic models. Our study is the first to catalog and characterize somatic mutations present in an animal model of genetic audiogenic epilepsy generated by artificial selection for seizure susceptibility over many generations, which results in high predisposition to epilepsy as occurs in the GASH/Sal model. Other animal models of audiogenic epilepsy generated by artificial selection include the GAERS [19], the WAG/Rij [20] and the WAR [23], in which an auditory stimulus triggers the onset of audiogenic seizures. In all these models, different types of studies on seizures and pharmacological treatments have been conducted in the inferior colliculus as an epileptogenic nucleus and in auditory pathways [31, 74–78]. In the GASH/Sal model, previous studies have determined that susceptibility to developing the epileptic phenotype shows an autosomal recessive inheritance pattern [24]. A recent study, which analyzed the transcriptome of the WAR model, highlighted changes in the expression of 64 genes after audiogenic stimulation, thereby identifying new genes involved in the epileptic phenotype [79]. However, the underlying genetic alterations of audiogenic susceptibility in these animal models remain completely unknown.

**Fig 8:**
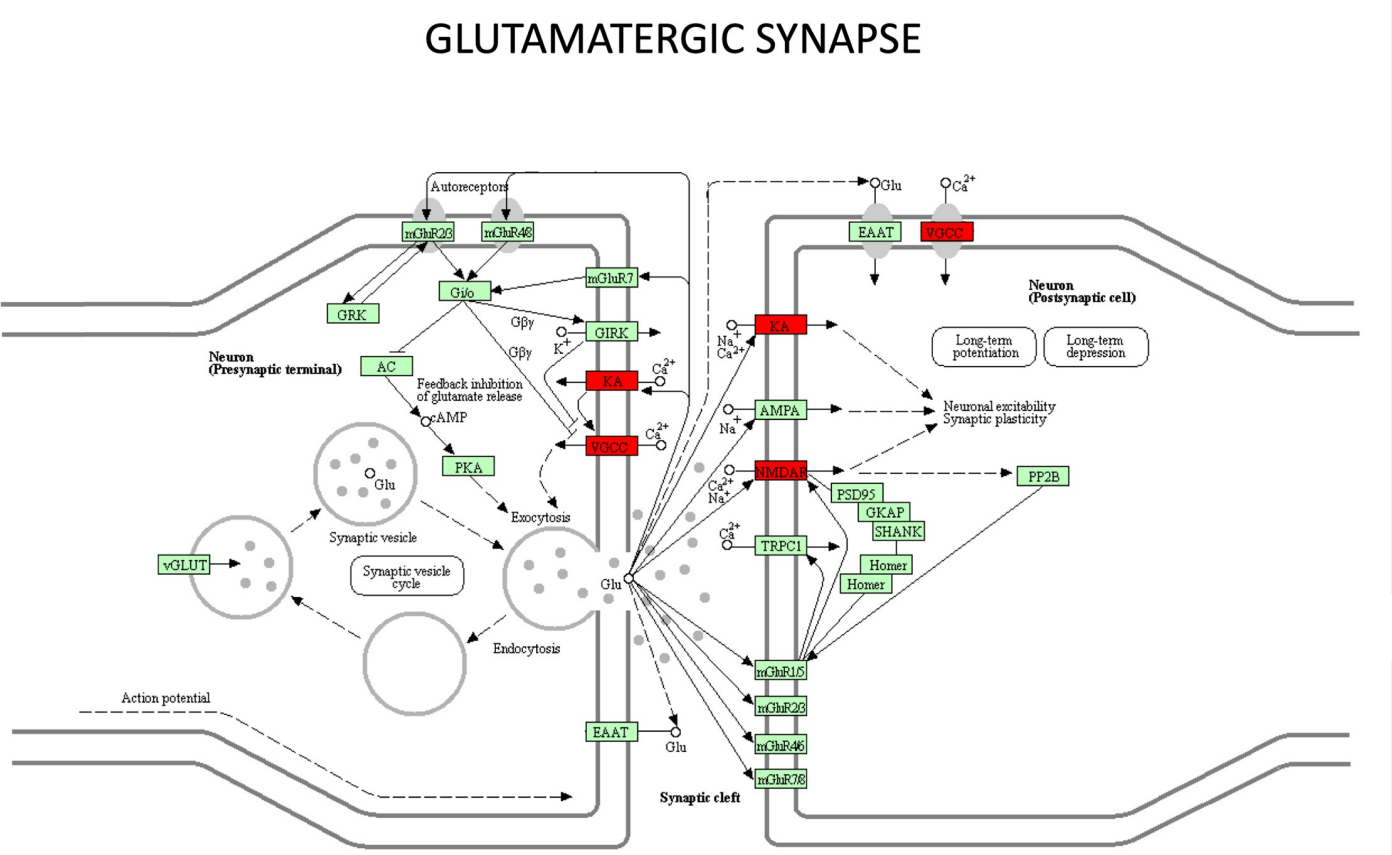
Glutamatergic synapse pathway. The components of the glutamatergic synapse pathways that are affected by “high-quality variants” are shown in red. KA is the glutamate receptor, ionotropic, kainate 4, and *Grik1* gene is the mutated component of this receptor. This receptor is found in both presynaptic, where it is activated by glutamate binding, thereby allowing Ca2+ influx, and postsynaptic membranes. VGCC is the voltage-gated calcium channel located in the presynaptic and postsynaptic membranes that is mutated in the *Cacna1a* gene, which encodes transmembrane pore- forming subunit of the P/Q-type or CaV2.1 VGCC, in the GASH/Sal hamster. Ca2+ influx through these channels is key to neurotransmitter release to the synaptic cleft. NMDAR is the N-methyl-D-aspartate receptor, another ionotropic glutamate receptor encoded by GluN1, located in the postsynaptic membrane (See https://www.guidetopharmacology.org/GRAC/ObjectDisplayForward?objectId=455). Together with the KA glutamate receptor, the control Na+ and Ca2+ influx, participating in postsynaptic depolarization. Therefore, two of the three ionotropic glutamate receptors of postsynaptic membranes are altered in GASH/Sal hamsters.

Our study nevertheless identifies high-impact variations in the *Asb14*, *Msh3* and *Arhgef38* genes, with important functional consequences in the resulting proteins, albeit with no known relationship with any type of epilepsy. However, we have found a set of moderate-impact variants of genes classically associated with different types of epilepsy, such as *Cacna1a*, *Zeb2* or *Grik1*. These genes, together with others with no known variants associated with some form of epilepsy to date either, including *Grin2c*, are involved in significantly enriched pathways directly related to neuronal excitability, such as the glutamatergic synapse pathway (Fig 8), and may therefore play a key role in the GASH/Sal phenotype. In this context, further studies must be conducted to confirm the involvement of these genes in the GASH/Sal phenotype. Our data, supported by previous findings [52], support that genetic analysis facilitate the identification of genetic biomarkers for the risk of seizures.

## Supporting information

S1 Fig Quality control of reads and samples. A) Density plot showing the average quality of the reads (Phred Score). B) Density plot showing the number of alignments per read on the genome. C) Density plot showing the distribution of distances between sequence pairs.
(TIFF)

S1 Table Quality analysis of the reads. (XLSX)

S2 Table Quality analysis of alignments. (XLSX)

S3 Table High impact variants. (XLSX)

S4 Table Moderate impact variants. (XLSX)

S5 Table Moderate impact variants by VarElect. (XLSX)
