# Correction: Analysis of gene variants in the GASH/Sal model of epilepsy

**DOI:** 10.1371/journal.pone.0231603

**Published:** 2020-04-03

**Authors:** Elena Díaz-Casado, Ricardo Gómez-Nieto, José M. de Pereda, Luis J. Muñoz, María Jara-Acevedo, Dolores E. López

In the funding statement, the grant number from the funder Spanish JCyL cofinanced with the European Union FEDER funds 2017 is listed incorrectly. The correct grant number is: SA070P17.
