# Supplementary figures and images for: Analysis of gene variants in the GASH/Sal model of epilepsy
Source: PLoS One. 2020 Mar 13;15(3):e0229953. doi: 10.1371/journal.pone.0229953 (PMC7069730; doi:10.1371/journal.pone.0229953)

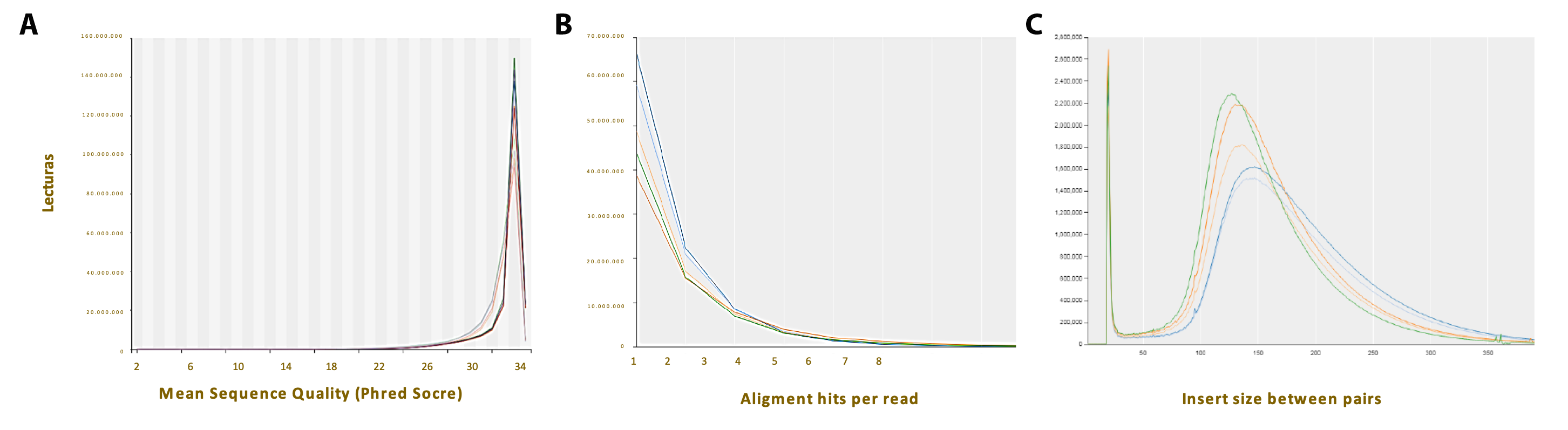

Supplement: S1 Fig — A) Density plot showing the average quality of the reads (Phred Score). B) Density plot showing the number of alignments per read on the genome. C) Density plot showing the distribution of distances between sequence pairs. (TIFF) [file pone.0229953.s001.tiff]
